# The Relationship between Diver Experience Levels and Perceptions of Attractiveness of Artificial Reefs - Examination of a Potential Management Tool

**DOI:** 10.1371/journal.pone.0068899

**Published:** 2013-07-23

**Authors:** Anne E. Kirkbride-Smith, Philip M. Wheeler, Magnus L. Johnson

**Affiliations:** Centre for Environmental and Marine Sciences, University of Hull, Scarborough, North Yorkshire, United Kingdom; Smithsonian’s National Zoological Park, United States of America

## Abstract

Artificial reefs are increasingly used worldwide as a method for managing recreational diving since they have the potential to satisfy both conservation goals and economic interests. In order to help maximize their utility, further information is needed to drive the design of stimulating resources for scuba divers. We used a questionnaire survey to explore divers’ perceptions of artificial reefs in Barbados. In addition, we examined reef resource substitution behaviour among scuba divers. Divers expressed a clear preference for large shipwrecks or sunken vessels that provided a themed diving experience. Motives for diving on artificial reefs were varied, but were dominated by the chance of viewing concentrated marine life, increased photographic opportunities, and the guarantee of a ‘good dive’. Satisfaction with artificial reef diving was high amongst novices and declined with increasing experience. Experienced divers had an overwhelming preference for natural reefs. As a management strategy, our results emphasize the capacity of well designed artificial reefs to contribute towards the management of coral reef diving sites and highlight a number of important areas for future research. Suggested work should validate the present findings in different marine tourism settings and ascertain support of artificial reefs in relationship to level of diver specialization.

## Introduction

Scuba diving is a burgeoning global activity with coral reefs being a major attraction to divers. As a niche market, recreational diving is widely acknowledged as being one of the tourism industry’s fastest growing markets [1]–[3], and as a consequence, many countries are establishing themselves as new international diving destinations. Coral reefs provide a diverse and stimulating setting for recreational diving, as well as other marine based activities. However, their ubiquitous appeal to the diving tourism industry has led to concerns of significant levels of biological damage resulting from the practice. Many studies have documented diver impacts [4]–[8], with levels of damage to reefs often linked to intensity of use by divers [9]–[11] and to a lack of diving experience [12]–[14]. Studies report; mechanical breakage [14]–[16] and the re-suspension of sediments [11], [16], [17] as problems.

Although there are negative impacts associated with mass diving tourism, scuba diving has the potential to generate substantial revenues [18]–[22]. However, balancing the requirements of reef conservation with the needs of local host economies represents a considerable challenge to managers and policy makers. Various approaches to manage coral reef diving sites have emerged over the previous 20 years, such as the percentile approach and limits of acceptable change [23], and the concept of ecological carrying capacity (e.g. [24], [25]). A drawback of these policies though, is that they may require ongoing monitoring and adjustments [26], and are more effective when applied within a marine park setting. Even within marine protected areas, active management is often lacking [27]–[30]. Artificial reefs could provide an alternative more unconventional method to assist in the management of scuba diving impacts.

Whilst artificial reefs are not viewed as ‘perfect’ substitutes for natural coral reefs [20], there is evidence that they are valued by scuba divers [31]–[33] with many structures used successfully as sacrificial dive sites worldwide [34]–[37]. Of significance, artificial reefs have been shown to alleviate user pressure to nearby natural reef habitats [34], [35], and to contribute substantially to local host economies [18]–[20], [34]. In view of the fact that some scuba divers place little importance on the ecological characteristics of a reef site [38], [39], it may be possible to satisfy divers’ requirements with well conceived artificial reef diving attractions. However, studies relating to issues concerned with the recreational use of artificial habitats by divers, have received scant attention to date. The few relevant published studies [32], [33], [40]–[43] and principal findings are presented in Table 1. A majority of these works sought to gain a personal insight from divers into their motivations and perceptions of diving on artificial reefs, but none investigated resource substitution behaviour among divers.

**Table 1:** Previous studies and key findings of motivational factors related to diving on artificial reefs.

	Milton, 1989 [40]	Stanley & Wilson,1989 [41]	Ditton et al. 2002 [42]	Stolk et al. 2005 [32]	Shani et al. 2011 [33]	Edney, 2012 [43]
Artificial Reef Attributes	Desirable fish species	Fish species (grouperand snapper)	Large Naval ships Petroleum structures	Old shipwrecks Diversity of species Concentration of marine life	Large Naval ships Airplanes Themed structures	Historical shipwrecks Artifacts Penetrable wrecks Marine life
Environmental Factors	Accessibility todive site	Underwater visibility	Mooring buoys Depth of reef	Sea visibility Currents Reef accessibility Reef location		
Social Factors	Travel time Previous experiences	Size of dive group	Restrictions on spear guns Night diving Tranquility Adventure	Size of dive group Safety Photographic opportunities		Peace Tranquility

The aim of this study was to explore the perceptions of diving on artificial reefs from a user perspective. Information was sought to characterize both resident and visitor scuba divers, to acquire an understanding of why individuals dive on artificial reefs, and the factors that inform their choice of dive site. We report on divers use, opinions, and preferences related to artificial reefs, including the environmental attributes and motivational factors that contribute to diver enjoyment. We also explore if reef habitat preference is influenced by diving experience. Our results are discussed within the context of scuba diving management where reef conservation is important.

## Methods

### Ethics Statement

All divers completed the survey themselves and gave their permission to use the results. Individuals were not identifiable from the data provided. The work described in this paper was reviewed and approved by the Centre for Environmental and Marine Science departmental ethics committee. Verbal assurance was provided by a representative of the Barbadian Coastal Zone Management Unit that no permit is required to conduct questionnaire based research on the island.

### Study Setting

**Figure 1:**
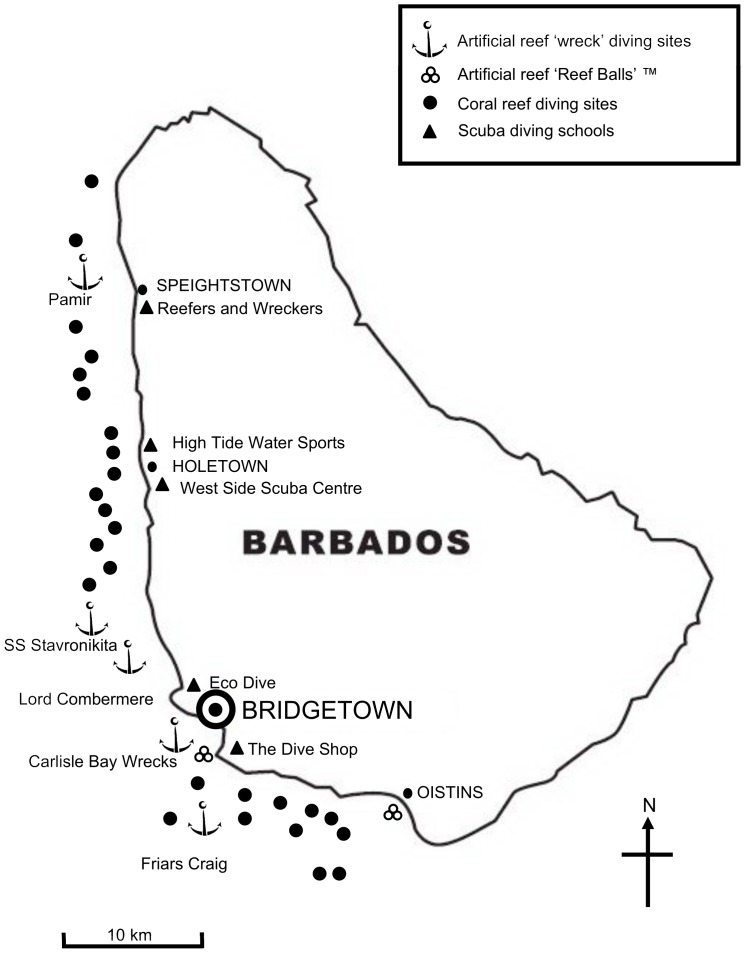
Map of Barbados. Locations of artificial reef and natural reef diving sites and diving schools.

The study was conducted on the Caribbean island of Barbados (13°10′N, 59°35′W), West Indies (Figure 1), between December 2010 to January 2012. Whilst Barbados is a relatively small island (431 km^2^), its current population of 276,300 [44] makes it one of the most densely populated islands in the Caribbean [45]. Along the protected western side of the island are complexes of fringing, patch, and bank reefs that nourish the white sand beaches [46]. These characteristics form the basis of the island’s tourism appeal [47] alongside warm tropical temperatures and clear marine waters. To complement the natural reefs, several artificial reefs consisting of shipwrecks and of Reef Balls™ (www.reefballs.org) have been gradually deployed along the south-west coast (Figure 1). Barbados has an extensive collection of wrecks [48], at various stages of maturity, six of which are situated in a dedicated marine park in Carlisle Bay. These factors, together with a diverse diving clientele [21] and the proactive attitude of the Barbados government towards artificial reefs [49], made this island an ideal site to conduct a study of the interactions of diving tourism and artificial reefs.

### Data Collection

Data was collected from a 36 question, self-administered survey (in English only), using a combination of open-ended and closed questions. The questionnaire was designed to profile the diving clientele of Barbados, report on their artificial reef awareness and use, their satisfaction of artificial reef diving, and their habitat preferences. A series of Likert scales (5–point) and checklists were included in the survey design alongside 8 free-response questions that gave individuals an opportunity to express their own thoughts and feelings to a prior response. Specific questions included in the survey reflect previous works that have studied diver perceptions of artificial reefs [32], [40], [42]. To assist participants; a map of Barbados was provided that included a list of all artificial reef and natural reef diving sites and locations of diving schools situated along the south-west coast of the island. Respondents were given the opportunity at the conclusion of the survey to add any additional information they thought necessary/beneficial to the study. Prior to the main survey, the questionnaire was tested as a pilot survey (*n* = 10) aided by a survey assessment sheet that resulted in minor modifications to several of the questions.

Sampling was conducted with the assistance of five of the diving companies situated along the south-west coast of Barbados (Figure 1). A twelve month survey period enabled us to capture one high season (November to May) and one low season (June to October). Selection of survey participants was randomized, based on every other individual entering a dive shop with the *a priori* requirement of ≥10 logged dives and knowledge of local artificial reef diving. The rationale of the study was made clear to all participants prior to completion of the questionnaire. Two hundred questionnaires were fully completed within the study period.

### Statistical Analysis

The Statistical Package for the Social Sciences (SPSS, Version 19) software was used to analyze relevant questionnaire data. For this study and consistent with the methodology of Fitzsimmons [39], a distinction was made between the experience level of divers; novice (<100 logged dives) and experienced (≥100 logged dives). Mean scores for factors such as age and length of diving career, were calculated. To assess the importance of artificial reef attributes presented in the survey, ranked lists of mean values were produced for both novice and experienced divers. We applied Chi-square tests (with Yate’s Continuity Corrections) to categorical variables to detect differences in responses to specific questions (i.e. dichotomous choice questions) and to attitude statements. We examined the relationship of responses to specific questions between diver experience categories and between genders. Content analysis was employed to analyze dominant themes relating to qualitative data. Significant contributions were extracted and presented within the discussion. As our data were not normally distributed (Kolmogorov-Smirnov Test), non-parametric statistical tests were applied. The non-parametric Kruskal-Wallis Test was used to compare diver experience in relation to artificial reef satisfaction scores. Additionally, a Mann-Whitney U Test was employed to analyze for differences relating to experience of divers and to reef habitat preference.

## Results

### Demographic Characteristics, Length of Stay, and Reasons to Visit

Of the 200 divers surveyed, the sample included more men (60.5%) than women. Collectively respondents averaged 43 years of age (±13.4 s.d.), ranging from 12 to 71 years. Fifty percent of those surveyed were British, 24.5% American, 15.5% Canadian, and 6.5% resided in Barbados. The remaining 3.5% of respondents were represented by three countries; Germany, Australia, and Bulgaria. The higher numbers of visitors from the United Kingdom and the United States are consistent with figures reported in a study conducted on Barbados [50] and with arrival data reported for the island generally [51]. With regard to the length of stay for non-resident respondents, the majority (43%) were visiting Barbados for 7–10 days duration followed by individuals staying for 14 days (24.5%). Cruise/day-trippers visiting the island accounted for 2.5% of those surveyed. For non-residents, the main reasons given for visiting Barbados were for either a general holiday (50%), or for a dedicated diving holiday (39%). A minority were visiting for the purpose of work or business (3%) or to visit friends or relatives (3%). Content analysis revealed the ‘other’ category (5%) mainly consisted of honeymooning couples (4%) or individuals on a golfing holiday (1%).

### Scuba Diver Experience

The diving experience of respondents was highly variable. A break down of diving qualifications held revealed that 66.5% possessed Open Water certification (basic and advanced level, CMAS*), followed by 27% of divers with Sport or Dive Master qualifications (CMAS***). The remaining participants were either Instructors (5.5%) or trainee divers (1%). To further assess each respondent’s level of diving experience, individuals were questioned on the number of dives they had logged. Respondents had logged an average of 190 (±264 s.d.) dives to date. Moreover, the study revealed novices, i.e. <100 logged dives, accounted for 52% of the sample (104 individuals), compared to 48% being experienced divers (96 individuals), with ≥100 dives logged. Five percent of participants (10 individuals) had logged 1000 dives or more in their diving history. An assessment of commitment to diving indicated a mean career length of 10.75 (±9.6 s.d.) years. One individual had been diving for 45 years on both reef types.

### Artificial Reef Awareness, Use, and Preferred Material

**Figure 2:**
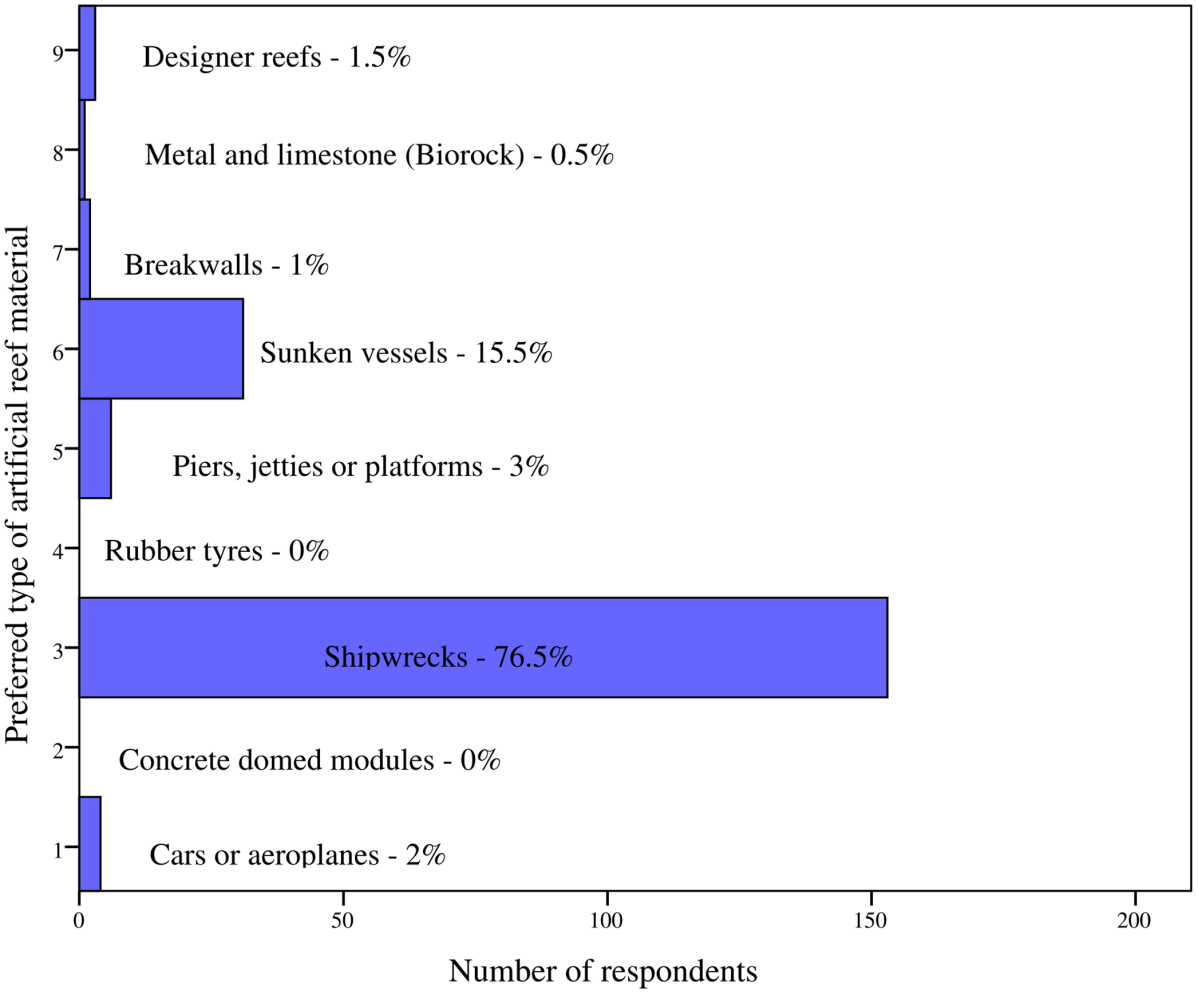
Respondents’ preferences for type of artificial reef material. Sample size: n = 200.

A number of exploratory questions were presented to participants to assess their awareness, use, and *a priori* knowledge of artificial reefs. Most divers (96%) had heard of the term ‘artificial reef’. As a reflection of this, 95% of respondents reported having previously dived on what they considered artificial habitat, whilst all 200 respondents had dived on artificial reefs in Barbados at some point. Divers were questioned on whether their decision to visit the island was influenced by established artificial reefs such as the group of six wrecks situated within Carlisle Bay. Twenty percent of individuals were found to be influenced by these reefs, and as such had chosen to visit the Caribbean island. When participants were asked if they had dived on these wrecks, 76% had done so. Respondents were also asked to state their most favoured type of artificial reef structure. From a list of 9 structures; 76.5% selected shipwrecks and 15.5% sunken vessels as their most preferred type. Figure 2 shows the least favoured structures consisted of rubber tyres (0%) and concrete domed modules (Reef Balls™) (0%). Despite the latter material receiving no support from divers, 12% of respondents had in fact dived on the conglomerate of Reef Balls™ deployed off the coastal area of Bridgetown (Figure 1). Divers were also asked to state their preferred depth at which to dive on artificial habitat. A majority (82%) selected having a preference for diving at less than 21 meters with only 2% of divers indicating a depth of more than 30 meters. The most favoured category was between 15–18 meters (38% of respondents).

### Satisfaction of Artificial Reef Diving

**Figure 3:**
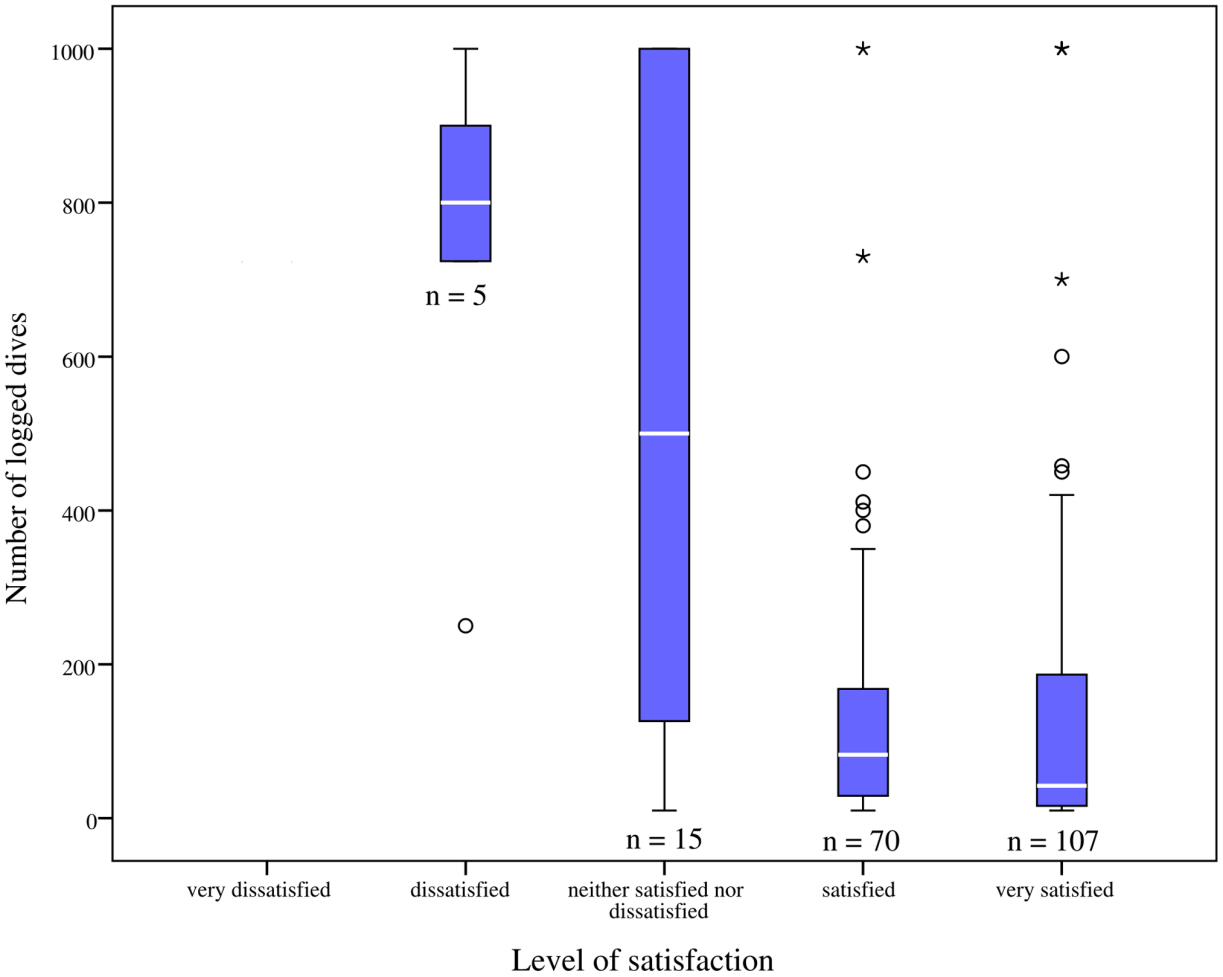
Divers’ satisfaction of artificial reef diving according to number of dives respondents had logged. Boxes represent the inter-quartile range that contains 50% of values. The median value is represented by a line across the box. The whiskers extend to the 5^th^ and 95^th^ percentiles and circles and stars outside the box plots are outliers. Sample size is represented by numbers below each box.

Analysis of responses to rate level of satisfaction (on a Likert scale of 1 to 5) to the experience of artificial reef diving in Barbados, revealed 90% of divers being either very satisfied (54%) or satisfied (36%) with the experience, while none reported being ‘very dissatisfied’. An exploratory analysis was conducted to assess any relationship between diver experience and level of satisfaction according to the number of dives respondents had logged (Figure 3).

A Kruskal-Wallis Test indicated a high level of association between diver experience and level of satisfaction relating to artificial reef diving (*x* ^2^ (3) = 23.90, *p*≤0.001) (Figure 3). This indicates that less experienced divers with fewer dives are significantly different from the experienced divers in rating their satisfaction. Post hoc analysis confirmed significant differences occurring between ‘very satisfied’ responses (*x* ^2^ (1) = 5.38, *p*≤0.020), and ‘neither satisfied nor dissatisfied’ responses (*x* ^2^ (1) = 6.67, *p*≤0.001), between novice and experienced divers. It appears therefore with increasing diving experience, level of satisfaction with artificial reefs as diving sites, decreases. Conversely, novice divers experience greater satisfaction with artificial reef diving. Analysis conducted to assess differences in diver satisfaction suggested no significant differences between males and females (*x* ^2^ (3) = 5.99, *p*≥0.112, *phi* = 0.174).

The next set of questions explored the level of importance of 13 artificial reef attributes that divers considered would enhance their diving enjoyment and satisfaction. Mean scores and overall ranking of reef attributes are presented in Table 2 for novice and experienced divers. Regardless of experience levels, respondents appear to derive a similar level of satisfaction from each of the attributes listed in Table 2. Ranked in the top six attributes for both diver groups are fish abundance, sea visibility, coral cover, safety, and reef colours. Fish abundance was significantly more highly ranked than reef complexity or reef size (*p*≤0.004 and *p*≤0.001, respectively). However, closer inspection of mean scores highlighted differences in ‘groups of attributes’ between levels of diver experience. For example, experienced divers placed greater importance on biological attributes including coral cover, reef colours, and reef complexity; whereas novices derived greater satisfaction from environmental attributes such as reef depth, location and access of reef, and sea visibility. Whist these latter results are not of statistical significance, further research to examine specific artificial reef attributes and their levels of importance to novice divers and experienced divers would be worthwhile.

**Table 2:** Ranked mean scores relating to the importance of artificial reef attributes for novice divers and experienced divers.

Overall rank	Novice divers (n = 104)		Experienced divers (n = 96)	
	Attribute	Mean score ±1SD	Attribute	Mean score ±1SD
1	Fish abundance	4.49±0.64	Fish abundance	4.51±0.68
2	Sea visibility	4.44±0.75	Sea visibility	4.40±0.77
3	Safety	4.28±1.09	Coral cover	4.38±0.74
4	Coral cover	4.11±0.84	Safety	4.32±0.97
5	Reef colours	4.01±0.92	Mooring buoys	4.12±0.98
6	Location/access	3.98±0.81	Reef colours	4.08±0.88
7	Mooring buoys	3.88±1.06	Location/access	3.96±0.86
8	Currents	3.74±0.85	Currents	3.72±0.86
9	Travel time	3.61±0.93	Reef complexity	3.62±0.85
10	Historic value	3.54±1.06	Travel time	3.59±1.03
11	Water depth	3.51±1.05	Water depth	3.46±1.09
12	Reef complexity	3.50±0.96	Historic value	3.44±1.09
13	Size of reef	3.34±0.86	Size of reef	3.34±0.92

Novice divers’ <100 logged dives, experienced divers’ ≥100 logged dives.

Values measured on a 1–5 point Likert scale: 1 = not important at all, 2 = not important, 3 = average, 4 = important, 5 = very important.

### Attitudes Towards Artificial Reefs

Respondents were presented with 8 attitude statements relating to artificial reefs that broadly addressed a number of ecological based themes. Table 3 presents each statement in rank order of divers’ agreement or disagreement. A majority concurred strongly with all five positively worded statements. The highest level of agreement provided was for the statement ‘artificial reefs provide new habitats for marine organisms’ with 93% of divers either agreeing (37%) or strongly agreeing (56%). Strong agreement was also recorded for the statement ‘artificial reefs take diver pressure off natural reefs’ with 81% either agreeing or strongly agreeing. As a reef management strategy, employing artificial reefs as alternative dive sites thus appears to have some resonance amongst divers surveyed in Barbados, as it has had elsewhere [32]. It appeared that many respondents considered ‘diving on an established artificial reef’ of no special interest compared to diving on a new artificial reef, with only 64.5% either strongly agreeing or agreeing, and a further 30.5% being ambivalent towards this statement. The neutral responses recorded may suggest that new, un-established artificial reefs are sufficiently attractive to some divers. There was a high level of disagreement (85.5%) towards the negatively worded statement ‘artificial reefs are a form of marine visual pollution’. When divers were examined on their attitude towards the statement ‘there are currently too many artificial reef dive sites in Barbados’, only a handful (4.5%) chose to agree with this statement.

**Table 3:** Divers’ ranked percentage agreement/disagreement to attitude statements concerning artificial reefs, with positively worded statements positioned at the top of the table, and values for the negatively worded statements below.

	*1	2	3	4	5	
Artificial reefs (AR)	(%)	(%)	(%)	(%)	(%)	Mean 1SD
Provide new habitat for organisms	0.0	0.5	6.5	37.0	56.0	4.49±.64
Take diver pressure off natural reefs	1.5	2.5	15.0	44.5	36.5	4.12±.86
Attract marine life divers wish to see	0.5	2.0	24.0	48.5	25.0	3.96±.79
Suitable substitute for diving	1.5	8.0	15.5	53.0	22.0	3.86±.90
Established AR are more interesting to dive	2.5	2.5	30.5	34.0	30.5	3.78±.96
Form of marine visual pollution	41.5	44.0	9.0	4.0	1.5	1.80±.87
Disruption to the local marine ecosystem	41.0	39.0	17.0	3.0	0.0	1.82±.82
Too many AR in Barbados	27.0	42.0	26.5	4.0	0.5	2.09±.86

* Values measured on a 1–5 point Likert scale: 1 = strongly disagree, 2 = disagree, 3 = neither agree nor disagree, 4 = agree, 5 = strongly agree. Sample size: n = 200 for each attitude statement.

### Opinions and Preferences: Artificial Reefs vs. Natural Reefs

For the final stage of the questionnaire, opinions and preferences relating to artificial reefs were sought in comparison to diving on natural reefs. Respondents were questioned on ‘whether they perceived artificial reef diving to be a nature-based experience, or not’. A high level of agreement (86%) was given in support of this question, which was found to be highly significant; *x* ^2^ (1) = 103.68, *p*≤0.001. When divers were asked if they agreed or disagreed with the question; ‘if there were aspects of diving on artificial reefs which are more satisfying when compared to diving on natural reefs’ 58% agreed; *x* ^2^ (1) = 5.12, *p*≤0.024. The relationships of responses to questions between diver experience categories and between genders were then examined.

Both novice (88%) and experienced (83%) divers agreed strongly that ‘artificial reef diving is a nature based experience’ (*x* ^2^ (1) = 0.706, *p*≥0.401, *phi* = 0.074). However, the second question asking ‘if there were aspects of diving on artificial reefs which are more satisfying’ indicated a significant difference in attitude between novices and experienced divers, with novices tending to agree more (*x* ^2^ (1) = 4.24, *p*≤0.039, *phi = *0.156). Moreover, there was a significant association between gender and responses to the latter two questions (*x* ^2^ (1) = 3.43, *p*≤0.044, *phi* = 0.151; *x* ^2^ (1) = 11.01, *p*≤0.001, *phi* = 0.258, respectively), with males being much more enthusiastic about artificial reefs in each case.

**Figure 4:**
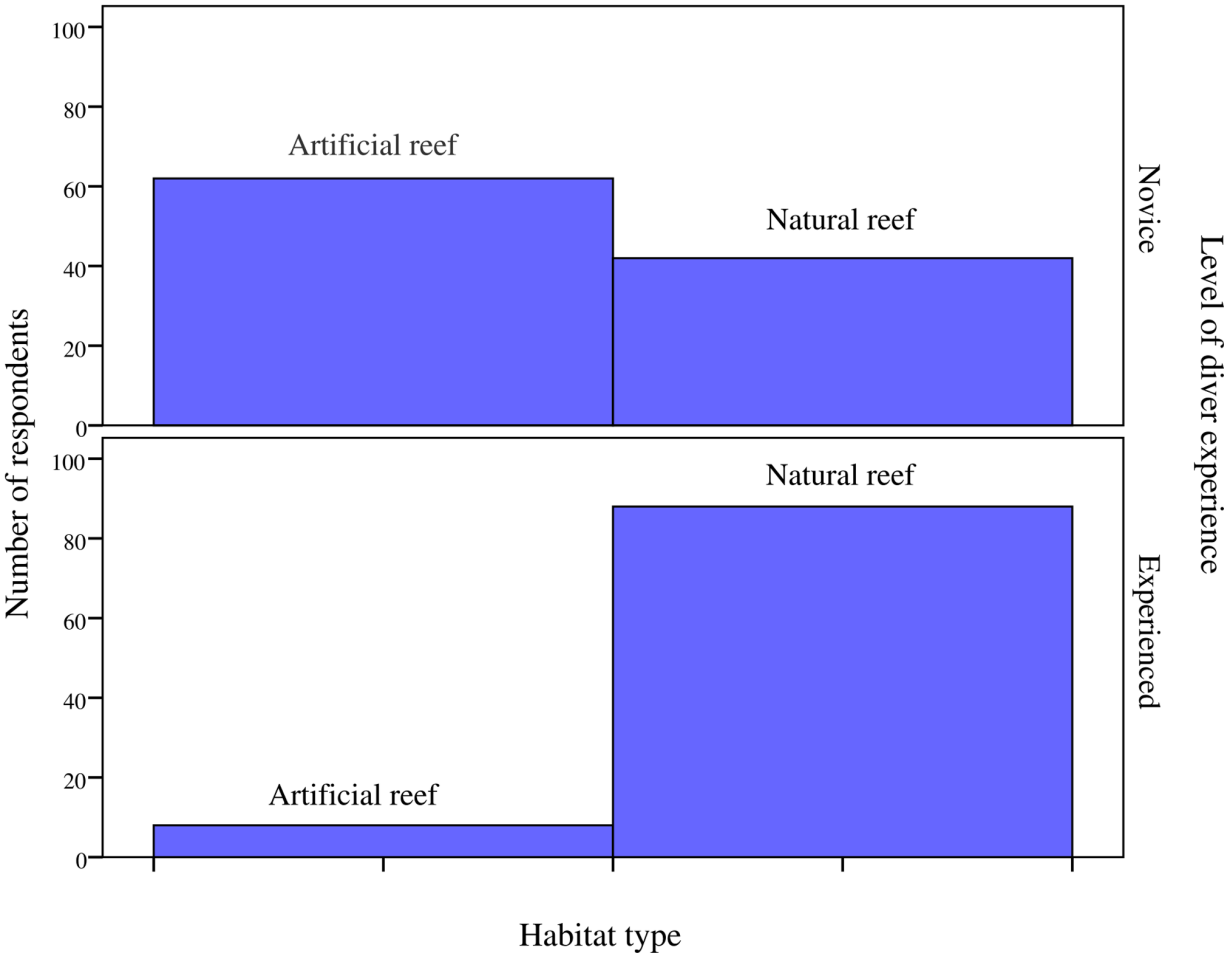
Preferences of divers for artificial and natural reef habitat types depending on level of diver experience. Sample size: novice divers (<100 logged dives) n = 104, experienced divers (≥100 logged dives) n = 96. Chi-square analysis to test for differences between divers choice of reef habitat; novice divers: *x* ^2^ (1) = 3.85, *p*≥0.062, experienced divers: *x* ^2^ (1) = 66.66, *p*≤0.001.

Respondents were also requested to state their preference for diving either on an artificial reef or on a natural reef in Barbados. Analysis revealed that a significant proportion (*x* ^2^ (1) = 18.00, *p*≤0.001) of divers chose natural reefs (65%). Differences between habitat preference and diver experience were explored. Figure 4 presents frequencies of responses for novice divers and experienced divers and their elected reef habitat. A Mann-Whitney U Test (2-tailed) revealed a highly significant difference in the number of dives logged and between respondents chosen habitat (*U = *2267, *z* = −5.848, *p*≤0.001, *r = *0.41). It is clear from Figure 4, that novice divers elected artificial habitat in preference to natural reefs, though a post-hoc analysis revealed no statistical difference between habitat choice (*x* ^2^ (1) = 3.85, *p*≥0.062); whist experienced divers had a strong preference for natural reefs as diving sites that revealed a highly significant result (*x* ^2^ (1) = 66.66, *p*≤0.001). However, no significant gender based association between these categorical variables was indicated (*x* ^2^ (1) = 0.913, *p*≥0.339, *phi = *0.078), despite females being less likely (30%) than males (38%) to choose artificial reefs for diving.

During the study period, divers interviewed were found to have performed a total of 1,280 dives. Of these dives, 57% (n = 729 dives) were conducted on natural reefs and 43% (n = 551 dives) on artificial reefs, revealing a highly significant difference between habitat use (*x* ^2^ (1) = 24.48, *p*≤0.001). In relation to diver experience; experienced divers performed significantly more (56%, n = 722) of these dives, in comparison to dives recorded (44%, n = 558) for novices (*x* ^2^ (1) = 20.76, *p*≤0.001). Experienced divers dived more on natural reefs (64%, n = 466 dives) than novice divers (36%, n = 263 dives) (*x* ^2^ (1) = 55.97, *p*≤0.001). In contrast, no apparent differences could be detected between experienced divers (46%, n = 256 dives) and novice divers’ (54%, n = 295 dives) use of artificial reef habitat (*x* ^2^ (1) = 2.62, *p*≥0.105).

## Discussion

In this study, the perceptions of scuba diving on artificial reefs in a tropical marine location were examined from a user perspective. The following discussion focuses on the main results and considers these findings in relation to improving and strengthening diving management and coral reef conservation.

### Characteristics of the Diving Clientele of Barbados

Recreational divers in Barbados broadly mirror the demographic profile of divers studied elsewhere in the world [2], [42], [52]. However, two points are worth noting. Our results confirm a general trend emerging in female acceptance of the sport [2], [52], [53], with almost 40% of divers surveyed being female. Divers sampled were older in comparison to other studies conducted worldwide (e.g. [32], [54]), though largely consistent with the findings of Schuhmann et al. [21] and Uyarra et al. [52] of divers previously studied in the Caribbean. These latter differences are likely to be a function of cost.

Novice and experienced divers were evenly represented in the study providing a diversity of views relating to artificial reef diving. Indeed, over half of all non-resident divers surveyed were return visitors, with some individuals having over thirty previous visits to Barbados. The study by Schuhmann et al. [21] revealed a similar trend in return visits, with half of their sample having had previous trips to the island. The provision of well conceived artificial reef diving sites, such as those situated within the Carlisle Bay area, appeared to influence the decision of some divers in this present study to visit Barbados.

### Artificial Reef Awareness and Preferred Material

Divers’ *a priori* knowledge and awareness relating to artificial reefs as diving resources was good. Most (96%) individuals surveyed had previously heard of the term artificial reef, and when invited to give an accurate description of this habitat, did so with accuracy. For example: *‘a structure placed on the seabed (intentionally or unintentionally) that attracts corals and associated marine species that over time appears natural’* (Participant 15), ‘*structures or objects deliberately placed in an accessible location for reef growth to be viewed by divers and snorkellers in the future’* (Participant 121). It was clear that embedded within a majority of the definitions was the perception that artificial reefs provide habitat for marine fauna and flora. In addition, the word shipwreck was used frequently in the descriptions provided by divers.

Shipwrecks and purposefully sunken vessels were identified as the most favoured artificial reefs to dive on by respondents. Consistent with our findings, divers studied by Ditton et al. [42], Stolk et al. [32], and Shani et al. [33] expressed an overwhelming preference for ex-naval ships, especially larger vessels that would absorb an entire dive. This latter point is of relevance to help ensure the success of artificial reefs in managing scuba diving. One of the primary goals of recreation-orientated artificial reefs is to generate ecological benefits [34] by diverting diving pressure from nearby natural reefs. Polak and Shashar [35] suggest the apathy among experienced divers to a new artificial reef in the Gulf of Eilat, Israel, was due in part to its modest size. In contrast, Dowling and Nichol [37] and Leeworthy et al. [34] reported positive environmental benefits surrounding the immersion of retired naval ships aimed at improving recreational diving in Western Australia and Florida respectively. It is clear, artificial reefs need to be substantial in size to avoid congestion, as this affects visitor satisfaction [54], [55] and depresses values [56].

### Satisfaction of Artificial Reef Diving

In general, divers were found to derive a high level of satisfaction from artificial reef diving in Barbados, with few individuals being dissatisfied or ambivalent (Figure 3). Stolk et al. [32] reported a strong level of satisfaction among a sample of Australian artificial reef divers, with their level of satisfaction changing with the type of artificial reef they dived on. When respondents were questioned on what they considered made an artificial reef satisfying to dive on, many divers agreed it was the ability to penetrate the larger wrecks that made a positive difference, as well as the wrecks historical connections and feelings of authenticity. A large number of divers additionally commented that bigger wrecks were preferred as they appear substantial enough to support a significant and complex ecosystem, providing diving motivation and satisfaction. Edney [43] recently studied diving motives specific to wreck divers in Chuuk Lagoon, Micronesia, and recorded participants as being focused on seeking out specific experiences, notably; historically significant wrecks, artifacts, the ability to penetrate wrecks, and the marine life encountered.

Despite such strong satisfaction reported for artificial reef diving, it was clear that as respondents experience increased, their level of satisfaction decreased (Figure 3). Dearden et al. [38] identified a general decline in diving satisfaction with increasing dive experience, with the authors concluding that more specialized divers, with a higher level of diving investment, tend to have more specific resource requirements than novice divers. Studies of other recreational activities (e.g. [57], [58]) additionally indicate a propensity for more specialized participants to have more specific resource requirements. Our findings thus suggest that less experienced divers may be more willing to support the use of artificial reefs as diving attractions.

When participants were asked to rate various reef attributes considered important to the enjoyment and satisfaction of diving on artificial reef habitat, the most valued characteristics for both novice and experienced divers were; fish abundance, sea visibility, safety, and coral cover. These results broadly reflect previous findings of attributes [32], [40], [41], [43] significant to artificial reef divers, and thus additionally confirm the importance of these features in contributing to the success of recreation-orientated artificial reefs. Clearly, there is general consensus that ‘fish’ are highly valued components of the diving experience (e.g. [2], [39]–[41], [52], [59]). Measures to therefore attract fish to reefs, either through the deployment of artificial reefs within marine protected areas, where fish abundance is often higher [60], or through the correct design of artificial reefs (e.g. [61], [62]), are crucial.

### Opinions and Preferences: Artificial Reefs vs. Natural Reefs

To develop a greater understanding of why divers choose artificial reefs as diving attractions, respondents’ personal experiences of artificial reef diving were sought relative to natural reef diving. Qualitative work by Stolk et al. [32] provided a basis for this. Many divers used artificial reefs due to the challenging nature of the dive, the themed experiences attached to shipwrecks and airplanes, and the overall guarantee of a ‘good dive’. In fact several individuals commented on artificial reef dives as being their most memorable. The concentration and diversity of marine life that artificial habitat attracts was mentioned frequently. Other salient elements of artificial reef diving discussed, revolved around their ease of access and increased photographic opportunities relative to natural reefs, the ‘uniqueness’ of the dive experience, the use of otherwise barren landscapes, and their ability to reduce diving pressure on natural reefs. This last point (both question and voluntary based) was made by a considerable number of respondents surveyed, as it was by Stolk et al. [32] Australian divers, thus reaffirming many divers active support of marine conservation [63].

Previous studies [18], [20], [64] have demonstrated that scuba divers place a greater value on diving natural reefs in preference to artificial reef habitats. Data from the present study supports these observations, as divers were generally found to have a significant preference towards the use of natural reef sites. This result is by no means surprising, despite many divers viewing artificial reef as a unique and stimulating type of diving experience [31]–[33]. However, it was clear that differences existed in respondents’ elected diving habitat. Whilst experienced divers chose natural reefs over artificial habitat (Figure 4), novices exhibited a greater preference for artificial reefs. Indeed, a recently deployed artificial reef in the Gulf of Eilat, Israel, was shown [35] to be effective in changing the behaviour of in-training and novice divers (but not of advanced divers), by reducing their use of nearby natural reefs. In view of the fact that novice divers (often with poor buoyancy control) are recorded as generally causing most damage to natural reefs [12]–[14], and represent a significant market share of the dive tourism market [65], these results have considerable implications for the management of scuba diving tourism.

### Artificial Reefs: Management Implications for Diving Tourism and Reef Conservation

Traditional practices aimed at controlling diver impacts on reefs have largely embraced the concept of ecological carrying capacity of divers (e.g. [24], [25]). However, the management of coral reefs necessitates more than this basic solution, it requires a range of tools, especially in non-reserve environments.

Considerable economic and ecological benefits can be achieved by developing diving destinations through the provision of artificial reefs for diving. In the first instance, they provide divers with a more diverse range of diving opportunities and environmental settings, essential factors in maintaining diving market interest and facilitating a competitive market edge [38]. In addition, Dearden and Manopawitr [66] predict that one possible effect of global climate change may be a reduced number of natural reefs on which to dive. Also, artificial reefs can act as dive training sites [35] providing divers with the opportunity to practice and develop their skills in less ecologically sensitive and hence more relaxed surroundings. This practice would reap ecological benefits, by removing more damaging in-training and novice divers from natural reefs [12]–[14]. Less degraded coral reefs would in turn attract experienced divers who place greater importance on the biological characteristics of a reef site [38], [39]. It is also increasingly appreciated that artificial reefs can serve as environmental educational tools as proposed by van Treeck and Eisinger [67].

In the absence of formal statistics for diving in Barbados, Schuhmann et al. [21] reported between 30,000 and 50,000 divers visiting the island per year. Using our data that suggests 2.75 dives on artificial reefs per visit, we can estimate that between 82,500 and 1.38 million dives take place on local artificial reefs per annum. Whilst these figures are encouraging, they may in part reflect the behavioral practice of local diving schools that often divide a two-tank dive between each habitat type (Personal observation), though some divers may request specific reefs to dive on (i.e. natural reefs only). For conservation reasons, the practice of diving schools routinely visiting both reef habitats per trip should be encouraged. Our results suggest that no significant loss in diver satisfaction would occur by using artificial reefs locally. Indeed, artificial reefs can in some instances be more popular than natural reefs, as other Caribbean diving destinations have recorded higher levels of artificial reef usage compared with natural reef dive sites. For example, in the British Virgin Islands, Hime [68] quoted diving figures for the Bow of the *RMS* Rhone as being 5,270 dives per year, representing four times as many dives undertaken in comparison to the busiest local natural reef.

In order to enhance current dive management practices using artificial reefs, the following points are recommended for consideration by marine resource managers and policy makers. Where artificial reefs are present: (1) transfer all introductory courses and in-training dives to artificial reef sites, (2) reinforce the environmental education of divers through the provision of educational materials positioned on artificial reefs, and (3) use more ‘in-depth’ conservation education dive briefings that have been shown [69] to reduce damage to reefs.

### Conclusions and further research

This study of artificial reef divers in Barbados contributes to the current body of knowledge (e.g. 32,33,40–43] and is useful to reef planners and marine tourism managers. Motives for diving on artificial reefs were dominated by the reliability of the diving experience and associated biodiversity viewing and wildlife photographic opportunities. Divers expressed a clear preference for themed diving experiences associated with large shipwrecks or sunken vessels.

Our findings show however, that our sample of divers is not homogenous - they differ in their satisfaction of artificial reef diving and reef habitat preference. Novice divers derive greater enjoyment and show a greater preference for artificial reef diving sites than their experienced diving counterparts. These findings therefore suggest that novice divers are more likely to accept reef habitat substitution more readily than experienced divers. To our knowledge, this study is the first to reveal recreation specialization in scuba divers relative to resource substitution behavior, and these results could have significant implications for the way reef based tourism is managed.

Further studies need to establish to what extent divers would support a reef substitution policy, as well as additional research to validate our present findings in different locations. Limited work exists in the field of diver specialization [38], and thus more in-depth studies would further identify differences in divers’ reef resource requirements using for example, a diver specialization index, such as the one constructed by Dearden et al. [38].
